# Not only phosphorus: dauciform roots can also influence aboveground biomass through root morphological traits and metal cation concentrations

**DOI:** 10.3389/fpls.2024.1367176

**Published:** 2024-05-24

**Authors:** Yulin Huang, Rong Fan, Xiaoqi Wang, Songlin Jiang, Wanting Liu, Wenli Ji, Weizhong Li

**Affiliations:** ^1^ College of Landscape Architecture and Arts, Northwest A&F University, Yangling, Shaanxi, China; ^2^ College of Forestry, Northwest A&F University, Yangling, Shaanxi, China

**Keywords:** dauciform roots, *Carex*, biomass, root morphology, phosphorus, metal cations

## Abstract

**Background:**

Phosphorus in the soil is mostly too insoluble for plants to utilize, resulting in inhibited aboveground biomass, while *Carex* can maintain their aboveground biomass through the presence of dauciform roots. However, dauciform roots lead to both morphological and physiological changes in the root system, making their primary mechanism unclear.

**Methods:**

A greenhouse experiment was conducted on three *Carex* species, in which Al-P, Ca-P, Fe-P, and K-P were employed as sole phosphorus sources. The plants were harvested and assessed after 30, 60 and 90 days.

**Results:**

(1) The density of dauciform roots was positively correlated with root length and specific root length, positively influencing aboveground biomass at all three stages. (2) The aboveground phosphorus concentration showed a negative correlation with both dauciform root density and aboveground biomass in the first two stages, which became positive in the third stage. (3) Aboveground biomass correlated negatively with the aboveground Al concentration, and positively with Ca and Fe concentration (except Al-P). (4) Root morphological traits emerged as critical factors in dauciform roots’ promotion of aboveground biomass accumulation.

**Conclusion:**

Despite the difference among insoluble phosphorus, dauciform roots have a contributing effect on aboveground growth status over time, mainly by regulating root morphological traits. This study contributes to our understanding of short-term variation in dauciform roots and their regulatory mechanisms that enhance *Carex* aboveground biomass under low available phosphorus conditions.

## 1 Introduction

Phosphorus (P), a crucial macronutrient, plays a vital role in various physiological and biochemical processes, and therefore in plant growth (Mollier and Pellerin, 1999; Bisson et al., 2017). While soil contains a substantial P reservoir, a major portion exists in stable forms such as aluminum (Al), calcium (Ca), or iron (Fe) phosphorus, rendering it unavailable to most plants (Hinsinger, 2001; Balemi and Negisho, 2012). To ease P deficiency, P fertilizers are commonly applied, however, only a fraction is absorbed by plants, with the rest swiftly binding to Al, Ca, or Fe in the soil, forming insoluble precipitates that lead to soil overnutrition (Raghothama and Karthikeyan, 2005; Richardson and Simpson, 2011). In addition, repeated P fertilization diminishes soil nutrient effectiveness, reducing the gains of plant productivity and aboveground biomass accumulation (Hou et al., 2020). Thus, it is crucial to optimize the utilization of insoluble P, prompting plants to develop various root traits.

The diverse root traits which plants developed are mainly divided into either “nutrient-foraging” traits or “nutrient-mining” traits (Shen et al., 2011). “Nutrient-foraging” traits are characterized by the changes in root morphology to increase absorption surface area, while “nutrient-mining” traits facilitate P solubilization through the exudation of carboxylates and protons (Wen et al., 2017; Becquer et al., 2021). In the case of Cyperaceae, they have their very own product, dauciform root (DR), an analogue of cluster root, in response to low P conditions (Lambers et al., 2006; Lambers, 2022). Dauciform root, a carrot-shaped structure with an enlarged axis and dense root hairs, mostly formed under low P conditions and can be inhibited by higher P supply (Shane et al., 2005). Even though dauciform roots differ in structure from the cluster roots of lupine, they share similar functions (Shane et al., 2005; Lambers et al., 2015). Under P deficiency, their specialized structure and physiological functions significantly increase the root surface area and enhance the secretion of acid phosphatases, carboxylates, and protons, facilitating efficient acquisition of insoluble P (Lamont, 1974; Lambers et al., 2006; Raven et al., 2018). The formation of dauciform roots was proven to promote aboveground biomass accumulation under certain stresses (Gusewell and Schroth, 2017; Fan et al., 2023a), the question here is, how?

The genus *Carex* is the largest genus in Cyperaceae, which can widely survive and thrive in barren and degraded meadows, playing an essential role both ecologically and economically (Gao and Yang, 2017; Liu et al., 2021; Fan et al., 2023b). When relying on insoluble P as the sole source, *Carex* exhibited higher biomass compared to the no-P treatments, showing its proficiency in insoluble P utilization (Pérez Corona et al., 1996). Even though the above-mentioned experiment did not consider the impact of dauciform roots, these roots are known for their ability to solubilize P, even outperforming mycorrhizae under low P conditions (Shane et al., 2006). So, is this the primary factor driving their aboveground biomass accumulation? Moreover, as the dauciform roots secrete carboxylates and protons, not only P is released but also the metallic cations such as Al, Ca, and Fe in the soil (Pérez Corona et al., 1996; Playsted et al., 2006). Under Al-P treatment, organic acids were released to ease Al toxicity, which can combine with carboxylates and organic anions to release P from the soil (Pérez Corona et al., 1996; Shane et al., 2008). Ca supply positively influences the length and density of lateral roots, with Ca^2+^ enhancing the secretion of endogenous hormones, and bolstering plant resistance to various stresses (Silva et al., 2001; Veatch-Blohm et al., 2023). Fe^3+^ can also be released by secretions through chelation and oxidation-reduction, proven to stimulate cluster root production (McCluskey et al., 2004; Marastoni et al., 2020). So, could the increased biomass of *Carex* grown in insoluble P be linked to the effects of these metal cations? Furthermore, dauciform roots, characterized by dense root hairs, directly lead to a specific root length (SRL) increase, which is a common mechanism to cope with P stress, reducing belowground inputs, and consequently boosting aboveground biomass (Shen et al., 2011; Becquer et al., 2021). So, to what extent does the morphological variation caused by dauciform roots contribute to the aboveground biomass accumulation?

As a quantification of plant morphological and physiological variations, traits reflect their diverse responsive strategies to environments. This study took a trait-based approach to explain the regulatory mechanism of dauciform roots on the *Carex* aboveground biomass in response to P stress and identify the primary factors influencing aboveground biomass. Three hypotheses were made to explore the pathways of dauciform roots in promoting aboveground biomass increase: (a) root morphology variation: dauciform roots with dense hairs extend the root length and specific root length, enhancing absorption efficiency and promoting aboveground biomass accumulation; (b) insoluble P dissolution and utilization: the exudation of dauciform roots dissolves insoluble P into an available form, promoting aboveground biomass accumulation; (c) metal cations effects: dauciform roots dissolve insoluble P and release specific metal cations, influencing aboveground biomass accumulation. These hypotheses aim to clarify the intricate processes behind dauciform roots’ impact on aboveground biomass under P stress.

## 2 Materials and methods

### 2.1 Experimental materials and overall design

Three *Carex* species (*Carex breviculmis*, *Carex giraldiana*, and *Carex filispica*), each capable of producing dauciform roots, were chosen for this experiment. The initial plant conditions were standardized, with each plant starting at 1.5 ± 0.3g fresh weight, 6 ± 1cm leaf length, 7 ± 2 leaf number, 4 ± 0.5cm root length, and 10 ± 3 main root number. They were planted in pots filled with 900g of quartz grains (mixture of 0-0.5mm and 0.5-1mm grains in a 7:3 volume ratio) in a greenhouse in Yangling, Shaanxi Province, China (108°4′31″E, 34°16′0″N), maintaining a temperature of 24-32°C, with artificial light supplementing natural light when cloudy (center wavelength of 589nm, red to blue ratio of 8.5:1, source height of 2m, the horizontal spacing of 2.5m).

Our experiment began in October 2022, including five treatments: P-deficiency (0-P), KH_2_PO_4_ treatment (K-P), AlPO_4_ treatment (Al-P), Ca_10_(PO_4_)_6_(OH)_2_ treatment (Ca-P), and FePO_4_ treatment (Fe-P). Of these, K-P served as a soluble P source, while Al-P and Fe-P represented major forms of microsoluble P in acidic soil, and Ca-P in alkaline soil. Before transplanting, all forms of P were added in powder and thoroughly mixed with the quartz grains at 50 mg kg^-1^, with 15 replicates per treatment and species. The 100-day experiment began with 10 days of P starvation, providing only pure water. Afterwards, plants were fed every 5 days to 80 percent of the field holding capacity of pots with the Hoagland nutrient solution (mmol·L^-1^: 2.5 Ca(NO_3_)_2_, 1 MgSO_4_, 0.02 EDTA-Fe, 0.009 MnCl_2_, 3×10^-4^ CuSO_4_, 7×10^-4^ ZnSO_4_, 0.04 H_3_BO_3_, 5×10^-4^ H_2_MoO_4_). The pH was maintained at 6.2-6.4, and KCl was used to ensure consistent potassium (K) levels among treatments.

### 2.2 Sampling and measurement

After 30, 60, and 90 days of treatments (hereafter described as the first, second, and third stages), three replicates per treatment per species were randomly chosen for harvest and measurements, respectively. During harvest, the aboveground parts of plants were separated at the surface of quartz, killed at 105°C for 30 min, dried at 70°C for 3 days to constant weight, and weighed for the aboveground biomass. The dried material was then ground and digested with a mixture of 4 mL concentrated HNO_3_ and 1 mL 30%v/v H_2_O_2_, after which, aboveground P concentration was measured by the Mo-Sb colorimetric method using Continuous Flow Analytical System (Flowsys) (Zhang et al., 2021) and Al, Ca, and Fe concentrations were measured using Flame Atomic Absorption Spectrometry (M410) (Jacques et al., 2007; Gou et al., 2020). All visible roots from each pot were collected, and after removing bulk soil, the rhizosphere soil was collected for analysis of available P concentration by the sodium bicarbonate extraction-molybdenum anti-spectrophotometry method (Hammel et al., 2000), and cleaned roots were observed against a dark background with a stereomicroscope (LECIA M165 FC), recording the number of dauciform root (with swollen axis and dense hairs) and calculating dauciform root density (number of dauciform roots/root biomass) (Masuda et al., 2020). The roots were then placed in water without overlap, and a Winrhizo scanner (resolution setting at 150-200 dpi) was used to capture images for analyzing root length (RL) and root volume (RV) of the entire root system. The roots were dried and weighed, and specific root length (SRL, m g^-1^, root length/root biomass) and root tissue density (RTD, g cm^-3^, root biomass/root volume) were calculated (Kang et al., 2022).

### 2.3 Statistical analyses

The analyses were performed using SPSS Statistics (ver 26.0, SPSS, Chicago, IL, USA) and the figures were produced using Origin 2022 (OriginLab Corp., Northampton, MA, USA). All data were tested for the homogeneity of variance, and DR density, root length, specific root length, and aboveground biomass data were log-transformed to meet the assumptions of linear models. Means and standard errors presented in tables were calculated from untransformed data. The difference in DR density among treatments was subjected to the Kruskal-Wallis test. One-way ANOVA was used to test for the differences of aboveground Al, Ca, and Fe concentration among treatments, followed by Fisher’s LSD *post-hoc* tests if differences existed. Partial correlation analysis examined relationships between DR density and aboveground biomass, rhizosphere available P, root morphological traits, and metal cations concentrations, controlling for time variables. Simple linear regression simulated the bivariate variable relationships between these traits, with correlation coefficients (*r^2^*) and significance levels (*p*) calculated. A structural equation model, performed by Amos 21.0 (Amos Development Co., Armonk, NY, USA), explored how dauciform roots influenced different traits affecting aboveground biomass accumulation.

## 3 Results

### 3.1 Dauciform roots

As can be seen in **Table 1**, different forms of P supply significantly affected dauciform root density. The K-P treatment showed no dauciform root formation throughout the entire stage, excluding it from subsequent analysis. Under the Al-P treatment, there were no differences among the three stages, while the DR density in the last stage was significantly lower than the other treatments. A consistent trend was observed in all the other treatments (except Al-P): DR density in the first two stages showed no significant difference, while the last stage exhibited a notable increase. **Table 2**, using partial correlation analysis to exclude the effects of time, reveals a positive correlation between aboveground biomass and DR density in all treatments except Al-P.

**Table 1:** Comparison of the density of dauciform roots (DR density) in the first (30 days), second (60 days), and third (90 days) stage after different treatments.

Stage	DR density (numbers g^−1^ DW)				
	0-P	Al-P	Ca-P	Fe-P	K-P
First	65.68 ± 33.88 c	8.43 ± 2.74 cd	178.12 ± 77.68 c	7.83 ± 4.04 cd	0.00 ± 0.00 e
Second	238.39 ± 118.47 bc	27.08 ± 13.20 c	46.58 ± 29.95 c	70.41 ± 32.90 c	0.00 ± 0.00 e
Third	468.60 ± 128.04 a	86.68 ± 13.03 c	457.85 ± 72.47 ab	497.97 ± 173.85 a	0.00 ± 0.00 e

DW, root dry weight. Values represent Means ± SE (standard errors). Different letters to the right of numbers indicate significant differences among groups (Kruskal-Wallis test, p<0.05).

**Table 2:** Relationships between the density of dauciform roots (DR density) and aboveground biomass under different phosphorus (P) treatments, using Partial correlation Analysis to exclude the effect of time.

Control variation			Aboveground biomass	
			*r*	*p*
Stage	DR density	0-P	0.423	0.031^*^
		Al-P	0.244	0.229
		Ca-P	0.449	0.021^*^
		Fe-P	0.453	0.020^*^

r and p represent the correlation coefficients and the levels of significance, respectively. ^*^ indicates significant correlation (p<0.05).

### 3.2 Root morphological traits

**Figure 1:**
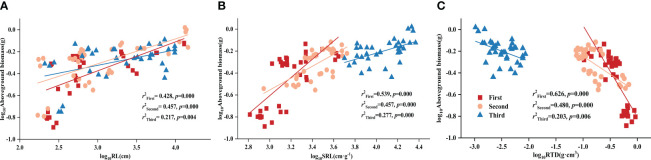
Relationships between **(A)** root length (RL) and aboveground biomass; **(B)** specific root length (SRL) and aboveground biomass; **(C)** root tissue density (RTD) and aboveground biomass in different stages. Simple linear regression was performed, and the red, orange, and blue lines represent the linear fits in three stages, respectively. *r^2^* values represent the correlation coefficients and *p* values represent the levels of significance (color printed).

Using partial correlation analysis to exclude the effects of time, RL and SRL both showed a positive correlation with DR density across various P treatments, while RTD showed a negative correlation with aboveground biomass (**Table 3**). What is noteworthy is that, in all three stages, all root morphological traits significantly influenced aboveground biomass: RL and SRL displayed positive correlations with aboveground biomass while RTD showed a negative one (**Figure 1**).

**Table 3:** Relationships between root length (RL), specific root length (SRL), root tissue density (RTD) and the density of dauciform roots (DR density), and aboveground biomass under different phosphorus (P) treatments, using Partial correlation Analysis to exclude the effect of time.

Control variation			DR density		Aboveground biomass	
			*r*	*p*	*r*	*p*
Stage	RL	0-P	0.728	0.000^*^	0.485	0.012^*^
		Al-P	0.648	0.000^*^	0.384	0.053
		Ca-P	0.782	0.000^*^	0.190	0.353
		Fe-P	0.850	0.000^*^	0.248	0.222
	SRL	0-P	0.761	0.000^*^	0.560	0.003^*^
		Al-P	0.822	0.000^*^	0.434	0.027^*^
		Ca-P	0.839	0.000^*^	0.199	0.330
		Fe-P	0.850	0.000^*^	0.316	0.116
	RTD	0-P	-0.193	0.346	-0.785	0.000^*^
		Al-P	0.138	0.503	-0.404	0.041^*^
		Ca-P	-0.279	0.167	-0.671	0.000^*^
		Fe-P	0.116	0.574	-0.760	0.000^*^

r and p represent the correlation coefficients and the levels of significance, respectively. ^*^indicates significant correlation (p<0.05).

### 3.3 Phosphorus concentration

**Figure 2:**
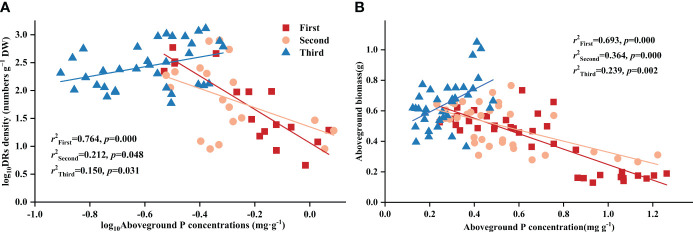
Relationships between **(A)** aboveground phosphorus (P) concentration and the density of dauciform roots (DR density); **(B)** aboveground phosphorus (P) concentration and aboveground biomass in different stages. Simple linear regression was performed, and the red, orange, and blue lines represent the linear fits in three stages, respectively. *r^2^* values represent the correlation coefficients and *p* values represent the levels of significance (color printed).

Throughout all three stages, DR density was significantly related to aboveground P concentration. Surprisingly, across all treatments, DR density showed a negative correlation with aboveground P in the first two stages, transforming into a positive one in the final stage (**Figure 2A**). The aboveground biomass and aboveground P concentration showed a correlation across all stages and treatments: after excluding the effects of time, aboveground biomass showed a consistent negative correlation with aboveground P concentration across all treatments (**Table 4**). As for the stages, aboveground biomass and aboveground P concentration showed a negative correlation in the first two stages, transforming into a positive one in the final stage (**Figure 2B**).

**Table 4:** Relationships between aboveground phosphorus (P) concentration and aboveground biomass under different phosphorus (P) treatments, using Partial correlation Analysis to exclude the effect of time.

Control variation			Aboveground biomass	
			*r*	*p*
Stage	Aboveground P concentration	0-P	-0.624	0.001^*^
		Al-P	-0.491	0.011^*^
		Ca-P	-0.699	0.000^*^
		Fe-P	-0.536	0.005^*^

r and p represent the correlation coefficients and the levels of significance, respectively. ^*^indicates significant correlation (p<0.05).

**Figure 3:**
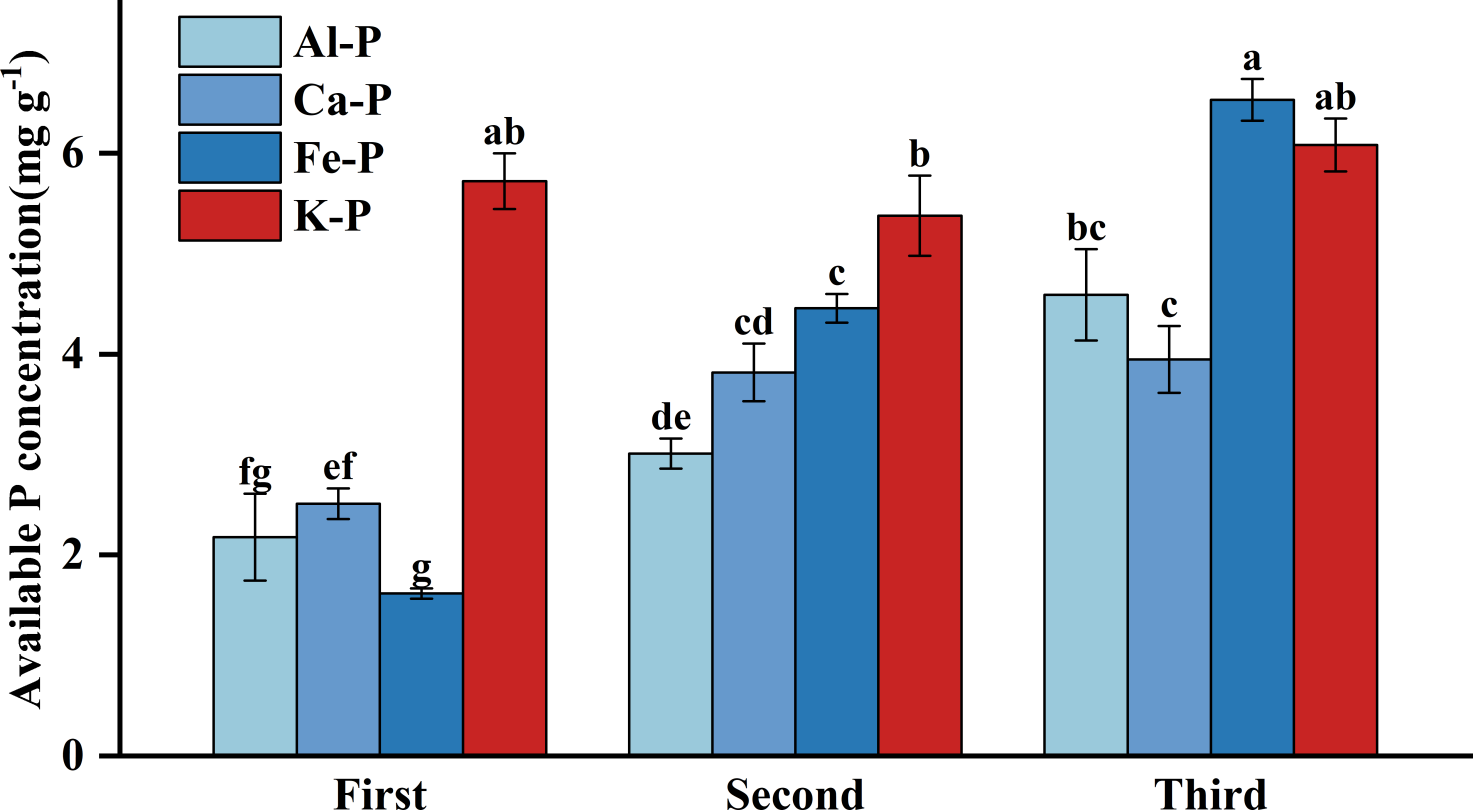
Differences in soil available phosphorus (P) concentration among different treatments in three stages. Bars represent Means ± SE (standard errors). Different letters above bars indicate significant differences among groups (LSD test, *p*<0.05) (color printed).

As **Figure 3** shows, there were significant differences in rhizosphere available P concentration among different treatments. Under Fe-P treatment, rhizosphere available P concentration was lower in the first stage yet significantly higher than all other insoluble P treatments in the final stage, even comparable to the K-P treatment. However, after the time-controlling by the partial correlation analysis, rhizosphere available P concentration showed no correlation with DR density in any treatment and was only negatively correlated with aboveground biomass under the Al-P treatment (**Table 5**).

**Table 5:** Relationships between soil available phosphorus (P) concentration and the density of dauciform roots (DR density) and aboveground biomass under different phosphorus (P) treatments, using Partial correlation Analysis to exclude the effect of time.

Control variation				DR density		Aboveground biomass	
				*r*	*p*	*r*	*p*
Stage	Available P concentration		Al-P	-0.214	0.293	-0.550	0.004^*^
			Ca-P	-0.092	0.657	-0.169	0.408
			Fe-P	0.334	0.095	0.051	0.806

r and p represent the correlation coefficients and the levels of significance, respectively. ^*^indicates significant correlation (p<0.05).

### 3.4 Metal cations

**Figure 4:**
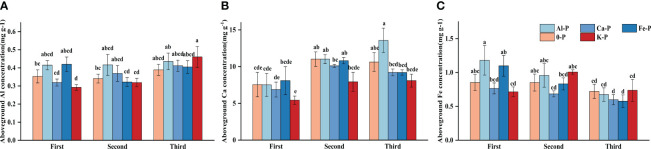
Differences in **(A)** aboveground aluminium (Al) concentration; **(B)** aboveground calcium (Ca) concentration; **(C)** aboveground iron (Fe) concentration among different treatments in three stages. Bars represent Means ± SE (standard errors). Different letters above bars indicate significant differences among groups (LSD test, *p*<0.05) (color printed).

In the final stage, there was a significant difference in Ca concentration between Al-P and K-P treatments, while Al and Fe concentrations showed no difference. All metal cation concentrations had no significant differences among treatments in the first two stages (**Figure 4**).

**Figure 5:**
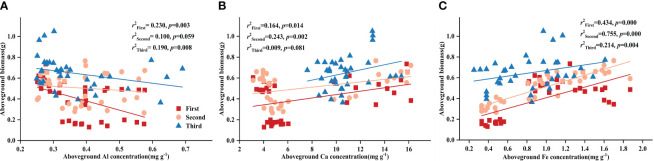
Relationships between **(A)** aboveground aluminium (Al) concentration and aboveground biomass; **(B)** aboveground calcium (Ca) concentration and aboveground biomass; **(C)** aboveground iron (Fe) concentration and aboveground biomass in different stages. Simple linear regression was performed, and the red, orange, and blue lines represent the linear fits in three stages, respectively. *r^2^* values represent the correlation coefficients and *p* values represent the levels of significance (color printed).

Controlling the time variables with partial correlation analysis, Fe concentration demonstrated significant correlations with both DR density and aboveground biomass in all treatments, except Al-P (**Table 6**). Additionally, all three metal cations (Al, Ca, and Fe) significantly influenced biomass accumulation in the Fe-P treatment (**Table 6**). Across all stages, aboveground biomass showed a negative correlation with aboveground Al concentration, but a positive correlation with Ca and Fe (**Figure 5**).

**Table 6:** Relationships between aboveground aluminium (Al) concentration, aboveground calcium (Ca) concentration, aboveground iron (Fe) concentration, and the density of dauciform roots (DR density) and aboveground biomass under different phosphorus (P) treatments, using Partial correlation Analysis to exclude the effect of time.

Control variation			DR density		Aboveground biomass	
			*r*	*p*	*r*	*p*
Stage	Al	0-P	-0.546	0.004^*^	-0.568	0.002^*^
		Al-P	-0.003	0.988	-0.037	0.858
		Ca-P	-0.343	0.087	-0.001	0.997
		Fe-P	-0.331	0.099	-0.402	0.042^*^
	Ca	0-P	-0.151	0.463	0.277	0.171
		Al-P	0.357	0.074	0.136	0.507
		Ca-P	0.078	0.706	0.266	0.189
		Fe-P	-0.076	0.712	0.521	0.006^*^
	Fe	0-P	0.446	0.022^*^	0.816	0.000^*^
		Al-P	0.198	0.332	0.356	0.075
		Ca-P	0.412	0.036^*^	0.423	0.031^*^
		Fe-P	0.433	0.027^*^	0.803	0.000^*^

r and p represent the correlation coefficients and the levels of significance, respectively. ^*^indicates significant correlation (p<0.05).

### 3.5 The structural equation modeling

**Figure 6:**
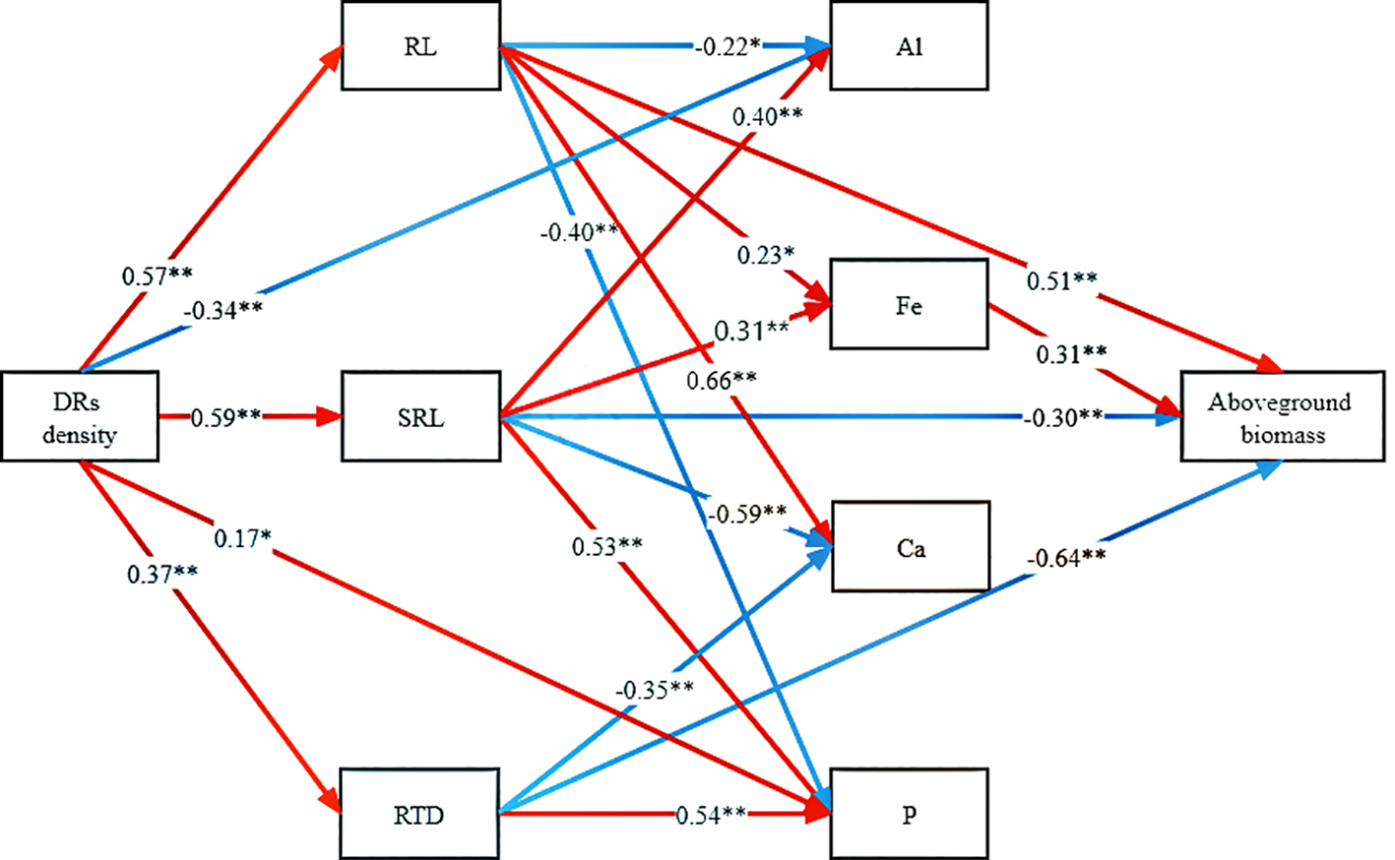
Path analysis of the direct and indirect effects of dauciform roots on aboveground biomass. DR density, the density of dauciform roots; RL, root length; SRL, specific root length; RTD, root tissue density; Al, aboveground aluminium concentration; Fe, aboveground iron concentration; Ca, aboveground calcium concentration; P, aboveground phosphorus concentration. The red and blue arrows represent positive and negative effects, respectively, and the numbers represent the standardized path coefficients (^*^ *p*<0.05, **p<0.01) (color printed).

To delve into the mechanisms through which DR density increased aboveground biomass accumulation, the linkages among traits were examined. DR density impacted RL, RTD, SRL, aboveground Al concentration, and aboveground P concentration. While RL, RTD, and SRL directly influenced aboveground biomass, RL and SRL also have an indirect effect through their impact on Fe concentration (**Figure 6**).

## 4 Discussion

### 4.1 The density of dauciform root

Among insoluble P treatments, DR density showed no significant difference over time in Al-P treatment, while Ca-P and Fe-P displayed significantly higher DR density in the final stage than the first two, correlating positively with aboveground biomass. On the one hand, this can be explained by that Al-P is the most utilizable form for plants, compared to the less available Ca-P and Fe-P, consistent with prior studies (Pérez Corona et al., 1996; Pearse et al., 2007; Zhang et al., 2011). On the other hand, the release of Al^3+^ from the dissolution of Al-P prompts plants to exude organic acids (citrate, malate, and oxalate, etc.) to avoid toxicity, which helps to mitigate P stress and greatly enhance P availability (Pérez Corona et al., 1996; Ma, 2007; Güsewell, 2016; Yan et al., 2021). Dauciform roots, highly sensitive to P supply, may be inhibited over time, by the high P availability in Al-P, resulting in the insignificant DR density differences in the final stage. In Al-P treatments, the mild P stress and a lower ratio of dauciform roots weight lead to their insignificant impact on aboveground biomass (Playsted et al., 2006; Han et al., 2022).

In contrast to Al-P, Ca-P and Fe-P are less soluble and therefore harder to utilize. Under P deficiency, plants undergo morphological changes, also release inorganic P from vacuole, and replace membranous phospholipids with non-phosphorus ones to maximize P utilizing efficiency (Lambers et al., 2006, 2012; Prodhan et al., 2019; Xu et al., 2019; Han et al., 2022). In the initial growth stages, plants can fulfill their needs with a small amount of P, which can be temporarily provided by their cells (Perkins and Lynch, 2021). In Ca-P and Fe-P treatments, DR density did not significantly differ in the first two stages yet increased in the third stage. In the beginning, smaller plants required less P, which could be easily met, explaining the lack of a significant response. It is noteworthy that aboveground biomass increased with increasing DR density, suggesting that, at a similar carbon cost, these *Carex* produce more dauciform roots in response to low P, rather than increasing root biomass allocation, allowing them to commit to aboveground investment (Güsewell, 2016). Previous studies indicated that under relatively low P supply, *Schoenus unispiculatus* decreased the root mass ratio while increasing aboveground growth (Shane et al., 2004). Meanwhile, the produced dauciform roots released insoluble P, alleviating P deficiency and enhancing aboveground biomass accumulation (Liu et al., 2022; Feng et al., 2023).

### 4.2 Effects of morphological traits

The abundant root hairs accompanying dauciform root production increased soil nutrient contact, increasing root length, and specific root length while decreasing root tissue density. Of these, RL reflects the space occupation of roots, directly influencing nutrient absorption capacity (Cheng et al., 2008). SRL and RTD, on the other hand, reflect nutrient absorption efficiency, with higher SRL signifying greater resource absorption per biomass unit, and lower RTD associated with enhanced nutrient effectiveness (Kramer-Walter et al., 2016; Miyatani et al., 2017; Bergmann et al., 2020). In our experiment, both RL and SRL increased with increasing DR density, which can be easily predicted and consistent with plants’ strategy to produce fine roots with higher SRL, reducing energy consumption for nutrient acquisition in P-deficient environments (Holdaway et al., 2011). This characteristic of dauciform roots promoted aboveground biomass accumulation, while RL and SRL showed a positive correlation with aboveground biomass across all treatments and stages. This suggests that *Carex* adopts a strategy of enhancing fine roots under P stress, expanding the contact area, maximizing soil nutrient utilization, and boosting aboveground biomass accumulation (Padilla et al., 2013; Fischer et al., 2018; Bakker et al., 2019, 2021), which has been verified in previous experiments on *Carex* (Pérez Corona et al., 1996).

### 4.3 Effects of P concentration

In the first two stages of this experiment, DR density showed no correlation with rhizosphere available P concentration, while exhibiting a negative correlation with aboveground P concentration, aligning with prior studies indicating that their formation is determined by aboveground P rather than soil P, increasing as aboveground P concentration decreases (Shane et al., 2005). However, in the final stage, DR density started positively correlating with aboveground P concentration, probably due to *Carex* with more dauciform roots efficiently acquiring insoluble P under extreme P-deficient conditions (Shane et al., 2006; Konoplenko et al., 2017; Fan et al., 2023a). Fe-P treatments had the highest rhizosphere available P concentration, owing to the highest DR density, which stimulated carboxylate secretion and enhanced soil P dissolution (Masuda et al., 2020). In the first two stages, aboveground biomass showed a negative correlation with aboveground P concentration, which might be related to the decrease in DR density as aboveground P concentration increased. However, in the final stage, both aboveground biomass and aboveground P were positively correlated with DR density, likely because the increased DR density promoted soil P dissolution, enhancing aboveground P concentration and biomass accumulation (Hou et al., 2020).

### 4.4 Effects of metal cations

The only difference in metal cation concentration among treatments is that Ca concentration in Al-P treatment was significantly higher than K-P in the final stage, which can be explained by the increased Ca^2+^ absorption to facilitate the secretion of organic acids, so as to alleviate Al toxicity (Silva et al., 2001; Sardans et al., 2023). As a crucial signaling molecule, Ca plays an essential role in mediating the response and adaptation of plants to environmental fluctuations by modulating the development of their roots (Yuan et al., 2021). An increase in Ca concentration not only promotes the exudation of organic acids such as citrate and malate but also enhances the length and density of lateral roots (Silva et al., 2001; Ma et al., 2014; Kochian et al., 2015), increasing plants’ resilience against various environmental stresses (Veatch-Blohm et al., 2023). In addition, Ca concentration can also influence the lamellar structures of chloroplasts, thus impacting the whole plant’s net carbon-assimilation capacity and the accumulation of aboveground biomass (Ding et al., 2018; Leitão et al., 2019), which is consistent with the positive relation between Ca concentration and aboveground biomass in our experiment.

Owing to the acid secretion mentioned above, even though the rhizosphere available P concentration showed a significant increase among stages in the Al-P treatment, there was no significant difference in Al concentration between Al-P and K-P treatments throughout the experiment, suggesting limited Al absorption. This limited absorption of Al could also be related to the presence of dauciform roots, which were proved to enhance the secretion of organic acids such as malate, particularly in early growth stages (Playsted et al., 2006). *Carex* species were found to release organic acids as chelating ligands to bind Al around their roots, which might be the way dauciform roots performed to weaken Al accumulation in the cell wall of roots to avoid physiological toxicity (Silva et al., 2018). Additionally, the promotion of the root metabolic reactions, such as the inward flow of H^+^ to increase the rhizosphere pH can also alter the form of Al around the roots, assisting *Carex* to resist Al toxicity by external exclusion and ease aboveground biomass inhibition (Ma, 2007; Yan et al., 2021).

Our experiment did reveal notable impacts of metal cations on biomass accumulation: Fe concentration showed a positive correlation with both DR density and aboveground biomass. Owing to the strong antagonism between P and Fe, P deficiency often enhances Fe accumulation, promoting the occurrence of lateral root primordia and the activation of auxin genes in their tips, stimulating lateral roots growth (Johnston et al., 2006; Giehl et al., 2012; Guo et al., 2022). Similar outcomes are observed in cluster roots, with a higher proportion in white lupin under Fe-P treatment (Shane et al., 2008). On the other hand, Fe was crucial for the electron transport components essential for photosynthesis and respiration, as well as the synthesis of enzymes essential for the tricarboxylic acid cycle, such as aconitase (de Vos et al., 1986; Zhang and Fernie, 2018). Through these ways, Fe stimulates chlorophyll production and influences the accumulation of photosynthetic products, thereby ensuring plant growth status and enhancing the accumulation of aboveground biomass (Chu et al., 2018; Marastoni et al., 2020).

### 4.5 Root morphological traits are the main predictors

Our experiments tested a series of hypotheses proposed, and structural equation modeling revealed that dauciform roots primarily contribute to aboveground biomass accumulation through variations in root morphological traits (RL, SRL, and RTD). This aligns with white lupin, where the dissolution of insoluble P depends mainly on root morphology and P effectiveness, exhibiting a location-specific pattern of P activation with higher acid phosphatase activity and lower pH closer to the root tip to promote the dissolution of insoluble P (Tiziani et al., 2020; Ma et al., 2021). Even though Fe also had a promoting effect on aboveground biomass, it was not directly influenced by dauciform roots, but was indirectly enhanced through their facilitation of specific root length. This suggests that the secretion of organic acids from dauciform root hairs did not significantly increase Fe uptake, while the increased specific root length along with the increased DR density played a role in enhancing absorption efficiency and facilitating Fe uptake, contributing to photosynthesis and aboveground biomass accumulation (Chu et al., 2018; Marastoni et al., 2020).

## 5 Conclusion

This study contributes to our understanding of short-term changes in dauciform roots under P stress, explains their advantage in insoluble P utilization, and explores the regulatory mechanisms enhancing the aboveground biomass of *Carex* under low P conditions. The experiment tested our hypotheses: Under different insoluble P treatments, the aboveground biomass of *Carex* increased with the increasing DR density, regulated by aboveground P rather than rhizosphere available P. Increased Fe uptake stimulated root growth and DR production, while Al uptake was inhibited to avoid toxicity, thereby increasing aboveground biomass. Furthermore, structural equation modeling verified that dauciform roots mainly contributed to aboveground biomass accumulation through direct root morphological changes and indirect promotion of Fe concentration by specific root length. However, a three-month experiment might not fully reveal long-term changes in dauciform roots and aboveground biomass accumulation, necessitating extended studies. Additionally, root secretions can directly reflect the physiological functions of dauciform roots and should be considered in further experiments.

## Data availability statement

The raw data supporting the conclusions of this article will be made available by the authors, without undue reservation.

## Author contributions

YH: Conceptualization, Formal analysis, Methodology, Writing – original draft, Writing – review & editing. RF: Conceptualization, Data curation, Software, Writing – review & editing. XW: Conceptualization, Data curation, Resources, Writing – review & editing. SJ: Conceptualization, Investigation, Methodology, Writing – review & editing. WTL: Data curation, Methodology, Writing – review & editing. WJ: Funding acquisition, Resources, Supervision, Writing – review & editing. WZL: Project administration, Supervision, Writing – review & editing.
